# Decentralized rare disease studies in Germany: first results and hurdles of secondary use of patient data

**DOI:** 10.1186/s12911-026-03688-7

**Published:** 2026-08-12

**Authors:** Michele Zoch, Christian Gierschner, Jens Weidner, Martin Sedlmayr, Gabriele Müller, Daniela Choukair, Georg F. Hoffmann, Nicole Toepfner, Reinhard Berner, Fabian Prasser, Josef Schepers, Helge Hebestreit

**Affiliations:** 1 Institute for Medical Informatics and Biometry, Faculty of Medicine and University Hospital Carl Gustav Carus, TUD Dresden University of Technology, Dresden, Germany; 2 Center for Evidence-Based Healthcare, Faculty of Medicine and University Hospital Carl Gustav Carus, TUD Dresden University of Technology, Dresden, Germany; 3 Department of Pediatrics and Center for Rare Diseases, University Hospital Heidelberg, Heidelberg, Germany; 4 Department of Pediatrics, Faculty of Medicine and University Hospital Carl Gustav Carus, TUD Dresden University of Technology, Dresden, Germany; 5 University Centre for Rare Diseases, Faculty of Medicine and University Hospital Carl Gustav Carus, TUD Dresden University of Technology, Dresden, Germany; 6 Medical Informatics Group and Medical Data Integration Center, Berlin Institute of Health (BIH) at Charité – Universitätsmedizin Berlin, Berlin, Germany; 7 Core Unit Digital Medicine and Interoperability, Berlin Institute of Health (BIH) at Charité – Universitätsmedizin Berlin, Berlin, Germany; 8 Center for Rare Diseases – Reference Center Northern Bavaria, University Hospital, Julius-Maximilians University, Würzburg, Germany

**Keywords:** Decentralized study, Health care process bias, Interoperability, Secondary data analysis, Rare diseases

## Abstract

**Background:**

The research challenges associated with rare diseases is characterized by a scarcity of information as well as reliable data due to their low prevalence. The problem of the “underpowered studies” is stemmed from a small research community, limited study participants and scarce data. The German project “Collaboration on Rare Diseases – Medical Informatics” (CORD-MI), tackles these problems by improving research opportunities and patient care by employing innovative IT solutions for collaborative data use across 20 German university hospitals. One possibility was to conduct decentralized studies based on secondary data. Three studies based on four rare diseases served as examples: (1) Cystic Fibrosis (CF), (2) Phenylketonuria (PKU), and (3) Kawasaki Disease and Multisystem Inflammatory Syndrome in Children (MIS-C).

**Methods:**

All three decentralized studies were conducted using routine inpatient data from German university hospitals. For each use case, an interdisciplinary team defined research questions, created analysis scripts based on the Core Data Set of the Medical Informatics Initiative (MII), executed these locally at participating sites, and aggregated anonymized results for descriptive statistical analysis. A frequency threshold rule was applied to protect patient privacy. Study protocols and analysis scripts for data extraction and evaluation are publicly available, and the studies were reported in detail in accordance with the Reporting of Studies Conducted Using Observational Routinely-Collected Health Data (RECORD) Statement.

**Results:**

Results from up to 17 German university hospitals were achieved for all three decentralized studies. The challenges in the areas of health care process bias, inaccurate coding of rare diseases, difficult verification of study results, and loss of information due to masking of small study case numbers as well as imprecise definition of the cohorts are discussed.

**Conclusions:**

Naming the hurdles enables the identification of areas for improvements, which will be the base for the development of new approaches or adaptations of existing tools and methodologies for the future. Although adaptation would make an impact, the initial results already show that decentralized analyses based on secondary use of patient data can improve research and thus also the care for people with rare diseases.

**Clinical trial number:**

Not applicable

**Supplementary information:**

The online version contains supplementary material available at 10.1186/s12911-026-03688-7.

## Background

Rare diseases are not only rare, but also come with rare information about them and few reliable data. Thus, the prevalence of a disease has a direct impact on the amount of research data, disease groups with lower prevalence are studied less frequently [1]. This problem of “underpowered studies” results in a small research community and small number of study participants. Finally, the lack of data makes rare disease research more difficult [2]. The use case “Collaboration on Rare Diseases – Medical Informatics” (CORD-MI) of the German Medical Informatics Initiative (MII) addresses this problem. The MII aims to improve research opportunities and patient care through innovative IT solutions that enable collaborative data use across institutions [3, 4]. It combines IT infrastructure development and scientific research projects. The project CORD-MI, which involved a total of 20 German university hospitals (of which 17 project partners took part in the study), pursued four goals: (1) improving the visibility of rare diseases, (2) providing insights into the reality of care, (3) improving the quality of patient care and (4) strengthening research in the field of rare diseases [5, 6].

Strengthening research on rare diseases can be achieved through the secondary use of patient data. Secondary health data analysis, which involves examining data primarily collected for a different purpose such as billing by health insurances, is a common tool in public health / health care research and epidemiology [7, 8]. In medical research, the focus has predominantly been on experimental studies, particularly randomized controlled trials (RCTs), and case-control studies [2]. Until now, the use of routine data for secondary data analysis has received limited attention. When secondary data has been analyzed, it has typically originated from registries or health insurances (e.g. [9, 10]). However, the potential to use secondary health care data provided by the health care facilities themselves offered enormous potential in terms of data flexibility, real-time assessment, etc. In the context of rare diseases, the potential benefits of secondary data use combined with multicenter data assessment is substantial [7, 8, 11]: These advantages include expanding the pool of potential study participants, achieving greater participant diversity due to geographic distribution, avoiding selection bias and the selective nonresponse of specific groups of individuals, and therefore reducing both costs and time.

As part of CORD-MI, several decentralized studies based on routine clinical data were conducted to explore the potential and possible obstacles associated with using secondary data for rare diseases. Decentralized studies are research models in which the collection of data is spread across numerous locations. Each individual institution analyzes the data locally using centrally designed scripts for data analysis, with only the (anonymous) aggregated results being shared. Consequently, this approach is designed to address privacy concerns and maintain data security. The methodological framework builds on an eight-step process for conducting decentralized secondary data analyses within the MII [12]. The following diseases served as examples: (1) Cystic Fibrosis (CF), (2) Phenylketonuria (PKU), and (3) Kawasaki disease and Multisystem Inflammatory Syndrome in Children (MIS-C).

1. CF is a congenital multi-organ disease with an incidence of about 1 : 3,300 to 1 : 4,800 in newborns in Germany [13]. Health improvements have led to more pregnancies in women with CF, but these pregnancies carry an increased risk for both mother and child [14]. Specialized centers, often located at university hospitals, are recommended for prenatal care and delivery in CF. In Germany, approximately 50 pregnancies per year were expected at the time this study was designed. The primary objective of the study was to determine the rate of deliveries in hospitals with a specialized CF center in relation to all deliveries in women with CF treated in these hospitals.
2. PKU is a congenital metabolic disease. Thanks to comprehensive newborn screening programs and optimized therapies, almost all patients with PKU are diagnosed in the first weeks of life and successfully treated in Germany since the late 1960s. The incidence in German-speaking countries is about 1:8,500 (on average) [15]. Despite most patients now being over 18 years old, adult medicine often lacks sufficient knowledge about PKU. Cross-sectional studies have indicated a clustering of relevant internal, neurological, and psychiatric symptoms in adults with PKU [16, 17], but larger or systematic studies are lacking.
3. Kawasaki disease is the most common form of generalized vasculitis in children aged 0 – 5 years with a yearly incidence of 7.2 : 100,000 in Germany in 2016 [18]. Due to its effects on coronary vessels, Kawasaki disease leads to significant morbidity and mortality. In recent years, more sensitive case definition and improved diagnosis have been developed [19]. In parallel with Kawasaki disease during the SARS-CoV-2 pandemic another, similar disease, Multisystem Inflammatory Syndrome in Children (MIS-C; synonym Pediatric Inflammatory Multisystem Syndrome (PIMS)), was observed. It was unclear whether SARS-CoV-2 infections equally contribute to Kawasaki disease and MIS-C and if the onset of Kawasaki and MIS-C vary by region, gender and age of the patients.

In the context of CORD-MI, the objective was to assess the suitability of the MII’s methods and infrastructure for addressing the specific challenges of rare diseases. After describing the general process to perform the distributed analyses [12], the next step was to investigate the quality and medical meaningfulness of the collected data. This study aims to give an insight into the results of the decentralized studies and to name hurdles that may arise when utilizing secondary data for rare diseases.

## Methods

The focus of this paper is on three use cases focusing on four rare diseases: (1) CF and delivery, (2) PKU, comorbidities in adulthood and pregnancy / delivery as well as (3) Kawasaki disease and MIS-C. The three use cases were selected as combinations of a rare disease and a specific complication or comorbidity, rather than the rare disease alone. This decision was informed by the following considerations, which were defined prior to data analysis: (a) the study was conducted exclusively at German university hospitals, where such complex clinical constellations are more likely to be treated, thereby increasing the likelihood of identifying relevant cases; (b) during the study period, the mandatory coding of rare diseases with ORPHAcodes in inpatient settings had not yet been introduced (spring 2023), so we selected complications that could be reliably identified using ICD-10-GM codes, which were available across all participating sites; and (c) the project’s medical experts aimed to evaluate whether the existing data availability and IT infrastructure were sufficient to reproduce established clinical knowledge for these conditions. This selection strategy ensured both data comparability across sites and the clinical interpretability of the aggregated results as well as clinical relevance.

The research questions of the medical use cases, which are specified in detail by primary and secondary questions, are listed in Table 1.

**Table 1:** Research questions of the medical use cases

Medical Use Cases	Primary Research Questions	Secondary Research Questions
(1) CF and pregnancy or delivery	(a) What was the percentage of pregnant women with CF who gave birth in a hospital with a CF center?	(b) How common were complications during these pregnancies and childbirths? (c) Which outcomes were observed in these children?
(2) PKU, comorbidities in adulthood and pregnancy or delivery	(a) How often were internal, neurological and psychiatric diseases documented in combination with PKU?	(b) Did internal, neurological and psychiatric diseases in PKU patients dependent on age? (c) How common were complications of PKU during pregnancy and childbirth? (d) Which outcomes were observed in the children of PKU women?
(3) Kawasaki disease and MIS-C	(a) How often were Kawasaki disease or MIS-C documented over time?	(b) How often were the diagnosis of Kawasaki disease or MIS-C documented in combination with COVID-19? (c) Did the frequency of Kawasaki disease or MIS-C diagnosis differ regionally in Germany? (d) Did the incidences of Kawasaki disease or MIS-C differ between genders and age groups?

All three studies were conducted following the eight-step process for decentralized secondary data analyses described in detail in Zoch et al. (2024) [12]. This process outlines a structured approach that includes initial study conception, protocol development, script creation, local execution at participating sites, aggregation of anonymized results, plausibility checks, iterative refinement, and final synthesis of findings. The previous study focused on technical infrastructure supporting this process, as well as the overarching meta-level perspective on its implementation from the standpoint of data scientists [12]. By referencing and adhering to this established process, the present study ensured methodological consistency while focusing on the medical content and interpretability of results. The same process was applied consistently across all three use cases to ensure methodological comparability.

The methodology of the present study is described in line with the Reporting of Studies Conducted Using Observational Routinely-Collected Health Data (RECORD) Statement [20]. Table 2 gives an overview of the RECORD items for each medical use case.

**Table 2:** RECORD items of the three use cases

RECORD Item	(1) CF, pregnancy and delivery		(2) PKU, comorbidities and pregnancy or delivery		(3) Kawasaki disease and MIS-C	
Study Design	The studies were conducted as descriptive studies to answer research questions on the basis of retrospective, observational data. Several sites (cross-sectional) were involved in these decentralized studies.					
Study Protocol	The study protocols are available on Zenodo (10.5281/zenodo.10656435 [21]).					
Setting						
Location	• Germany • 17 university hospitals		• Germany • 17 university hospitals		• Germany • 14 university hospitals	
Observation Period	2015–2021		2015–2021		2015–2021	
Participants						
Eligibility Criteria	• Inpatient care • CF:		• Inpatient care • PKU:		• Inpatient care • Kawasaki disease:	
	**ICD code**	**Name**	**ICD code**	**Name**	**ICD code**	**Name**
	E84.*	Cystic Fibrosis	E70.0	Classical phenylketonuria	M30.3	Mucocutaneous lymph node syndrome [Kawasaki disease]
			E70.1	Other hyperphenylalaninaemias	• MIS-C:	
					**ICD code**	**Name**
					A48.3, R57.8, R65.*	Toxic shock syndrome, other shock, Systemic Inflammatory Response Syndrome [SIRS]
					D76.1–4	Other specified diseases with participation of lymphoreticular and reticulohistiocytic tissue
					D89.8/9	Other specified disorders involving the immune mechanism
					U10.9	Multisystem inflammatory syndrome associated with COVID-19, unspecified
Variables						
Scripts	• [22]		• [23]		• [24]	
Codes	• Delivery		Comorbidities:		COVID-19 finding:	
	**ICD code**	**Name**	**ICD code**	**Name**	**ICD code**	**Name**
	O09.*	Duration of pregnancy	N18.*	Chronic kidney disease	U07.1	COVID-19, virus identified
	O24.*	Diabetes mellitus in pregnancy	F32.*	Depressive episode	U07.2	COVID-19, virus not identified
	O30.*	Multiple gestation	F33	Recurrent depressive disorder	U07.3	Personal history of COVID-19, unspecified
	O63	Long labour	F34	Persistent mood [affective] disorders	U07.43	Post COVID-19 condition, unspecified
	O64.*	Obstructed labour due to malposition and malpresentation of fetus	G31.0/9	Other degenerative diseases of nervous system, not elsewhere classified	U07.53	Multisystem inflammatory syndrome associated with COVID-19, unspecified
	O75.*	Other complications of labour and delivery, not elsewhere classified	Pregnancy / delivery:		U08.9	Personal history of COVID-19, unspecified
	O80	Single spontaneous delivery	**ICD code**	**Name**	U09.9	Post COVID-19 condition, unspecified
	O81	Single delivery by forceps and vacuum extractor	O09.*	Duration of pregnancy	U10.9	Multisystem inflammatory syndrome associated with COVID-19, unspecified
	O82	Single delivery by caesarean section	O24.*	Diabetes mellitus in pregnancy		
	P05.*	Slow fetal growth and fetal malnutrition	O30.*	Multiple gestation		
	P07*	Disorders related to short gestation and low birth weight, not elsewhere classified	O63.*	Long labour		
	P95	Fetal death of unspecified cause	O64.*	Obstructed labour due to malposition and malpresentation of fetus		
	Z37.*	Outcome of delivery	O75.*	Other complications of labour and delivery, not elsewhere classified		
	Z38.*	Liveborn infants according to place of birth	O80	Single spontaneous delivery		
			O81	Single delivery by forceps and vacuum extractor		
			O82	Single delivery by caesarean section		
			P05.*	Slow fetal growth and fetal malnutrition		
			P07*	Disorders related to short gestation and low birth weight, not elsewhere classified		
			P95	Fetal death of unspecified cause		
			Z37.*	Outcome of delivery		
			Z38.*	Liveborn infants according to place of birth		
Data Sources	The studies were based on routine data from hospital information systems at German university hospitals. These were prepared as Fast Healthcare Interoperability Resources (FHIR) for secondary use of patient data in accordance with the data and information model of the core data set of the German MII [12, 25]).					
Bias	The routine data of the university hospitals were not restricted. No other data sources (e.g. data outside of university hospitals, like registers) were taken into account.					
Study Size	• Routine data as part of the secondary data utilization of 17 German university hospitals		• Routine data as part of the secondary data utilization of 17 German university hospitals		• Routine data as part of the secondary data utilization of 14 German university hospitals	
Quantitative Variables	• Frequency threshold rule with threshold of 5		• Frequency threshold rule with threshold of 5 • Grouping in age groups: ◦[0,18], [19,99] ◦[0,5], [6,15], [16,18], [19,99]		• Frequency threshold rule with threshold of 5 • Grouping in age groups: ◦[0,17], [18,30], [31,99]	
Statistical Methods	The study comprises quantitative evaluations based mainly on descriptive methods. The results data from the individual locations were aggregated by combining them in a database at the data management center. Structured Query Language (SQL) scripts were used to calculate totals and counts.					
Data Access and Cleaning	No public databases were used. The raw data are available in the data integration centers of the participating university hospitals. It is in the nature of decentralized analyses that no access to the raw data is granted. No data cleaning methods were used.					
Linkage	No record linkage was performed. The anonymous results data from the individual locations were aggregated by transferring them to a joint results database.					

1 The inclusion criteria were described using codes of the German Modification of International Statistical Classification of Diseases and Related Health Problems in the 10th Version (ICD-10-GM)

2 ICD code valid since 2021

3 ICD code only valid in 2020

A team of data scientists created analysis scripts with *R*, which are based on the common data and information model of the MII – the MII Core Data Set (MII-CDS) [25, 26]. These scripts were then distributed so that each study site could carry out the analyses locally and only had to send back the anonymous results data. The scripts are available in a separate Git repository:

1. CF, pregnancy and delivery [22],
2. PKU, comorbidities and pregnancy / delivery [23] as well as
3. Kawasaki disease and MIS-C [24].

Due to data availability (e.g. not all data providers were able to access vital signs from neonatology information systems), only certain research questions could be answered. Table 3 provides an overview.

**Table 3:** Overview of research questions (numbering corresponds to Table 1) that could be answered by the developed scripts ( = addressed,  = basis for further consideration,  = no answer possible (e.g. due to missing data))

Medical Use Cases	Research Questions			
	(a)	(b)	(c)	(d)
(1) CF and pregnancy or delivery				-
(2) PKU, comorbidities and pregnancy or delivery				
(3) Kawasaki disease and MIS-C				

To protect privacy in the results, cell suppression with a frequency threshold rule with threshold of 5 was implemented [25]. Thus, the determined frequencies were combined categorically: [0] [1, 4], from 5 the determined frequency. The category [1,4] does not allow any statement to be made about the absolute number of patients, a value range for the maximum is specified by multiplying the number of listed [1, 4] categories with 4. In the following the results of this category were called “censored results”. The third category [5,∞] indicates the actual results that were equal or greater than 5. These values were referred to below as “absolute values”. In order to be able to show a trend despite the masking of the small values, the absolute value was added once with the minimum possible value of the censored results and once with the maximum possible value of the censored results, so that a value range could be determined.

The data were analyzed using descriptive statistics. For data presentation, results from decentralized studies were combined by adding numbers provided from each of the hospitals contributing data. For the category [1, 4], two approaches were taken: (1) the lowest number of ‘1’ was used for all respective hospitals reporting this category to provide an estimate of the minimum total number, and (2) the highest number of ‘4’ was chosen to estimate the maximum total number.

The study was conducted in accordance with the relevant ethical guidelines. An informed consent was not obtained because the study was based on the analysis of secondary data without any direct intervention or impact on patients. The result data were used in an anonymized form, ensuring the protection of identities of patients. From a legal perspective, the requirements for informed consent did not apply due to established data protection laws: (1) Under German Federal Data Protection Law (Bundesdatenschutzgesetz, BDSG), the processing of personal data for scientific research and statistical purposes is permitted but must be conducted in an anonymized form (§ 27 “Data processing for scientific or historical research purposes and for statistical purposes”). (2) The General Data Protection Regulation (GDPR) does not apply to anonymized data, as stated in Recital 26 (“No application to anonymized data”). Additionally, data protection officers were consulted at most study sites to ensure compliance with data protection regulations. The study was approved by the Ethics Committee of the University Hospital at the Julius-Maximilians-University Würzburg (reference number: EK 50/20), the Ethics Committee of the University Hospital Carl Gustav Carus at the TUD Dresden University of Technology (reference number: BO-EK-420092020) and the Ethics Committee of the University Hospital Heidelberg (reference number: S-292/2020). Other study sites either obtained an individual vote from their local ethics committee or followed the lead vote of the University Hospital at the Julius-Maximilians-University Würzburg.

In addition to ethical and data protection consultations, further key aspects were considered to ensure compliance with best practices and transparency. This included adherence to the guidelines on the processing of health data for research purposes of the European Data Protection Board [27] and on the reuse of health data for research purposes of the World Health Organization [28]. Furthermore, project details were described by following the RECORD statement and made publicly available to enhance transparency and reproducibility.

## Results

The results obtained from the decentralized studies are presented below in accordance with the predefined research questions. Each study is described separately, stating the observation period, primary and secondary outcomes and aggregated results.

### Use case (1): CF, pregnancy and delivery

The results of the study on CF with additional diagnoses related to pregnancy or childbirth are based on observations from the period of 2015 to 2021. The primary outcome is the number and distribution of such patients, while secondary outcomes include the distribution by pregnancy duration categories, delivery modes, and selected obstetric complications. Across the observation period, the data show consistently low counts of such cases, with occurrences documented across multiple pregnancy durations, delivery modes, and types of obstetric complications.

#### CF, pregnancy and delivery (to answer research question 1a and 1b)

**Fig. 1:**
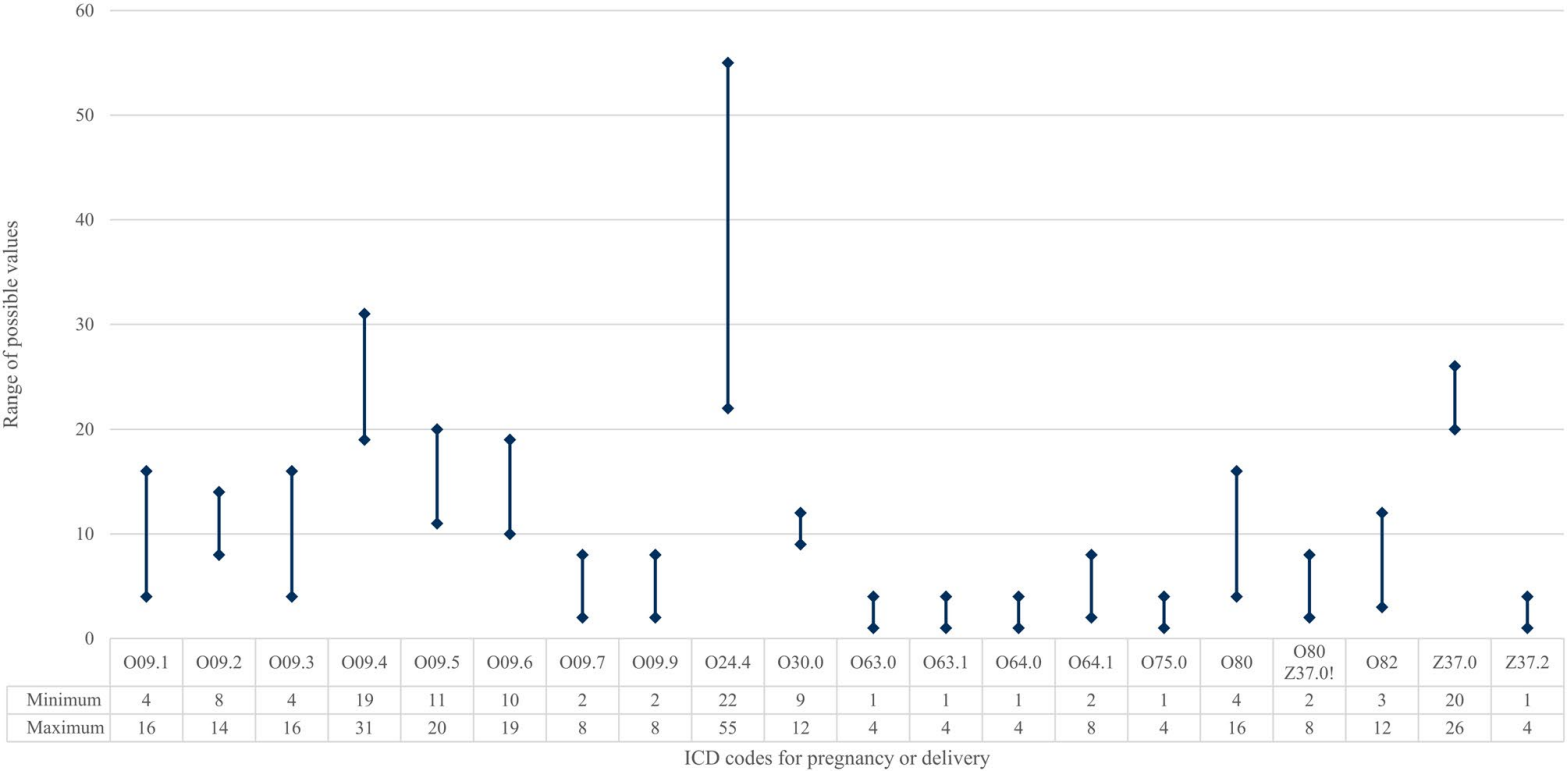
Range of possible values of patients with cystic fibrosis (CF) and ICD-10-GM diagnoses indicating pregnancy or childbirth, based on aggregated anonymized inpatient data from participating German university hospitals, 2015–2021. ICD-10-GM codes: O09.1 = Duration of pregnancy: 5th to 13 completed weeks, O09.2 = Duration of pregnancy: 14th to 19 completed weeks, O09.3 = Duration of pregnancy: 20th to 25 completed weeks, O09.4 = Duration of pregnancy: 26th to 33 completed weeks, O09.5 = Duration of pregnancy: 34th to 36 completed weeks, O09.6 = Duration of pregnancy: 37th to 41 completed weeks, O09.7 = Duration of pregnancy: more than 41 completed work, O9.9 = Duration of pregnancy: unspecified, O24.4 = Diabetes mellitus arising in pregnancy, O30.0 = twin pregnancy, O63.0 = prolonged first stage (of labor), O63.1 = prolonged second stage (of labor), O64.0 = obstructed labor due to incomplete rotation of fetal head, O64.1 = obstructed labor due to breech presentation, O75.0 = maternal distress during labor and delivery, O80 = spontaneous vertex delivery, O82 = Single delivery by caesarean section, Z37.0 = Single live birth, Z37.2 = twins, both liveborn

In the observation period, 86 to 118 deliveries in participating hospitals by women with CF were identified (see Table 1 (additional file)). Figure 1 shows the number of women with CF in combination with ICD codes that indicate delivery.

The most common complication (see Table 2 (additional file)) was gestational diabetes (*n* = 25–52). There were also isolated cases of obstructed labor due to malposition and malpresentation of fetus (*n* = 3–12) or other complications of labor and delivery, not elsewhere classified (*n* = 3–12).

### Use case (2): PKU, comorbidities and pregnancy or delivery

The study on PKU, conducted over the observation period 2015–2021, had as its primary outcome the range of patient counts (minimum and maximum) for each documented comorbidity. Secondary outcomes addressed differences between comorbidities and the occurrence of pregnancy- or childbirth-related diagnoses in women with PKU. The data show documented comorbidities in PKU patients with notable differences in patient counts between comorbidity groups, with depressive disorders being the most frequently recorded psychiatric comorbidity. Small numbers of PKU patients with pregnancy- or childbirth-related diagnoses were observed in selected years, with cases recorded across different pregnancy durations and delivery modes.

#### PKU and comorbidities (to answer research questions 2a and 2b)

**Fig. 2:**
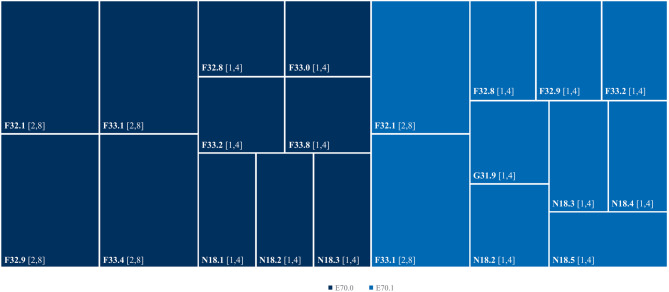
Minimum and maximum number of patients with phenylketonuria (PKU) and documented comorbidities, based on aggregated anonymized inpatient data from participating German university hospitals, 2015–2021. ICD-10-GM codes:. F32.1 = moderate depressive episode, F32.8 = other depressive episodes, F32.9 = depressive episode, unspecified, F33.0 = recurrent depressive disorder, current episode mild, F33.1 = recurrent depressive disorder, current episode moderate, F33.2 = recurrent depressive disorder, current episode severe without psychotic symptoms, F33.8 = other recurrent depressive disorders, G31.9 = degenerative disease of nervous system, unspecified, N18.1 = chronic kidney disease, stage 1, N18.2 == chronic kidney disease, stage 2, N18.3 = chronic kidney disease, stage 3, N18.4 = chronic kidney disease, stage 4, N18.5 = chronic kidney disease, stage 5

The Treemap in Figure 2 shows the number of censored results for comorbidities in PKU patients (see Table 3 (additional file)) for two different diagnosis codes E70.0 (Classical PKU) and E70.1 (Other Hyperphenylalaninaemias). Psychiatric comorbidities appeared in both diagnosis codes; these included depressive episodes and recurrent depressive disorders. Occasionally, chronic kidney diseases were detected. Overall, the number of patients with documented comorbidities was very low. This is in line with the low frequency of PKU patients with comorbidities relative to the total number of PKU patients (2.15%).

When looking at the relative frequency of PKU associated with internal, neurological and psychiatric diseases depending on the age group (see Table 4 (additional file)), it becomes clear that most comorbidities occurred in the age group of 31–99. The patients have depressive episodes or depressive disorders as well as chronic kidney diseases.

#### PKU and pregnancy or delivery (to answer research question 2c)

There is little information on birth complications in PKU patients. The results show that gestational diabetes and other complications such as obstructed labor due to fetal position, posture and attitude abnormalities occurred in 2 to 8 deliveries (see Table 5 (additional file) and Table 6 (additional file)).

### Use case (3): Kawasaki disease and MIS-C

The study on Kawasaki disease and MIS-C covers the observation period 2015–2021. The primary outcome is the annual count of patients for each condition, while secondary outcomes include age- and sex-specific distributions and the occurrence of COVID-19-related diagnoses in 2020–2021. The data show Kawasaki disease cases in all years of the observation period and MIS-C cases in 2020–2021, with distributions reported by age group and gender. Co-occurrence with COVID-19-related diagnoses was observed for MIS-C, and male patients were more frequently affected.

#### Kawasaki disease and MIS-C over time (to answer research question 3a)

**Fig. 3:**
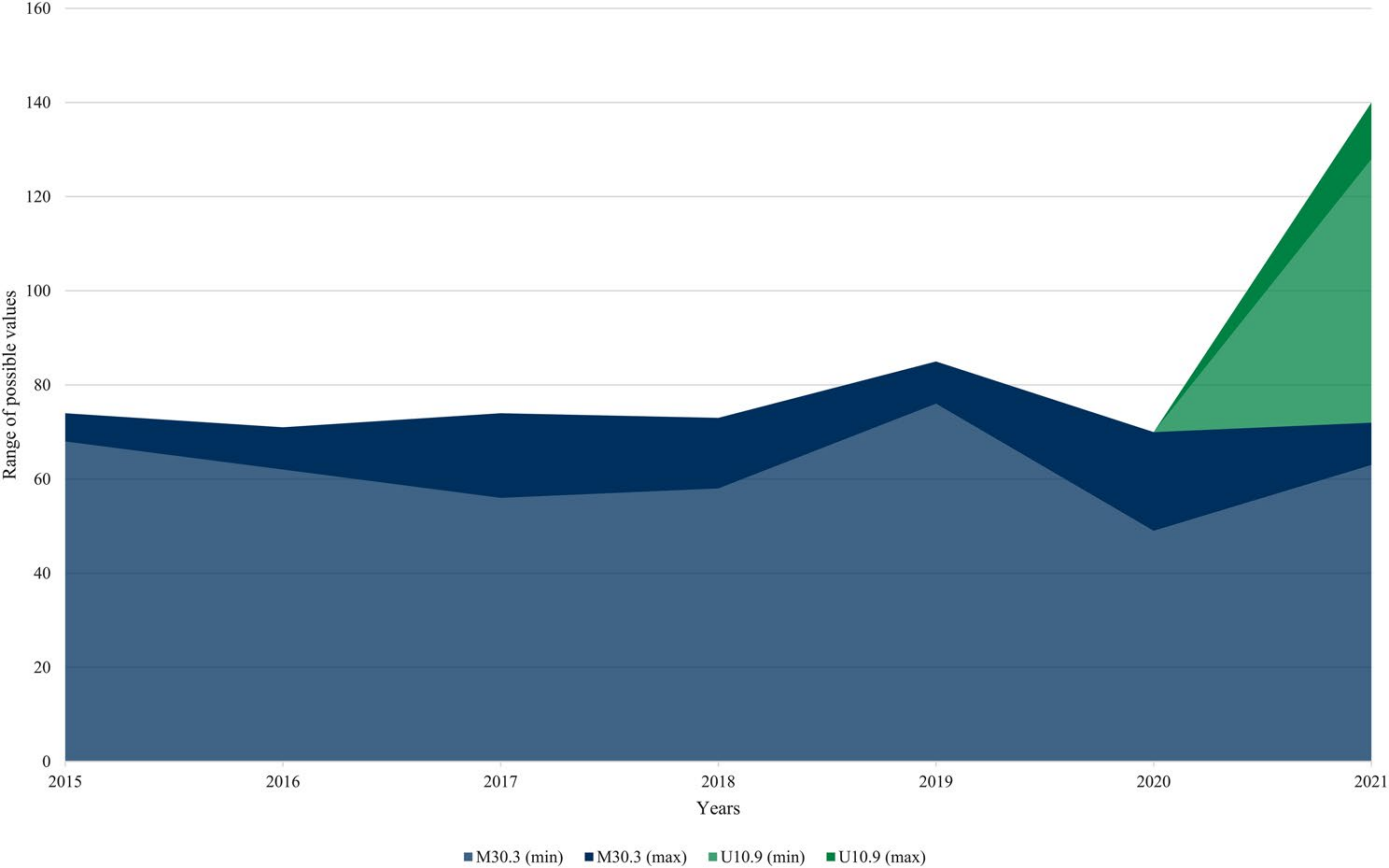
Range of possible values of patients with Kawasaki disease or Multisystem Inflammatory Syndrome in Children (MIS-C), based on aggregated anonymized inpatient data from participating German university hospitals, 2015–2021. ICD-10-GM codes: M30.3 = Kawasaki disease, U10.9 = MIS-C

Over time, there was a slight decrease in children with Kawasaki disease diagnosed in 2020 and a very sharp increase in children with MIS-C in 2021 (see Fig. 3; Table 7 (additional file)). It should be noted that MIS-C could only be coded with an independent ICD code since 2021: The code U10.9 stands for “Multisystem inflammatory syndrome associated with COVID-19, unspecified” and includes (a) “Kawasaki disease-like” syndrome temporally associated with COVID-19, (b) Multisystem inflammatory syndrome in children (MIS-C) temporally associated with COVID-19, (c) Pediatric inflammatory multisystem syndrome (PIMS) temporally associated with COVID-19 and (d) Cytokine storm temporally associated with COVID-19. Kawasaki disease (ICD code: M30.3) is explicitly excluded from U10.9.

#### Kawasaki disease and MIS-C in combination with a positive COVID-19 diagnosis (to answer research question 3b)

In order to be able to assess whether patients with Kawasaki disease or MIS-C also have a previous history of positive COVID-19 diagnosis, the corresponding diagnostic codes (only regarding the COVID-19 diagnosis) were searched for:

- Positive finding:
  o U07.1 – COVID-19, virus identified
  o U07.3 (in 2020) and U09.9 (in 2021) – Personal history of COVID-19, unspecified
  o U07.4 (in 2020) and U09.9 (in 2021) – Post COVID-19 condition, unspecified

- Negative finding:
  o U07.2 – COVID-19, virus not identified

- Inflammatory syndrome:
  o U07.5 (in 2020) and U10.9 (in 2021) – Multisystem inflammatory syndrome associated with COVID-19, unspecified

Information on COVID diagnosis was not available for all patients with Kawasaki disease and MIS-C, which is why the values in Table 4 only refer to cases with one of the above mentioned diagnoses. However, when looking at the combination of Kawasaki disease and MIS-C with a positive COVID diagnosis (see Table 4), it became clear that the vast majority of MIS-C patients had a positive COVID diagnosis. This could not be determined so clearly for patient with Kawasaki disease.

**Table 4:** Kawasaki disease and MIS-C diagnoses associated with one SARS-CoV-2–related diagnosis, based on aggregated anonymized inpatient data from participating German university hospitals, 2020–2021

Diagnosis	COVID-19 finding	2020		2021	
		Minimum	Maximum	Minimum	Maximum
**Kawasaki disease**	Positive Finding	0	0	13	37
	Negative Finding	2	8	1	4
	Inflammatory Syndrome	1	4	23	47
**MIS-C**	Positive Finding	-	-	33	90
	Negative Finding	-	-	2	8

#### Kawasaki disease and MIS-C depending on gender (to answer research question 3d)

**Fig. 4:**
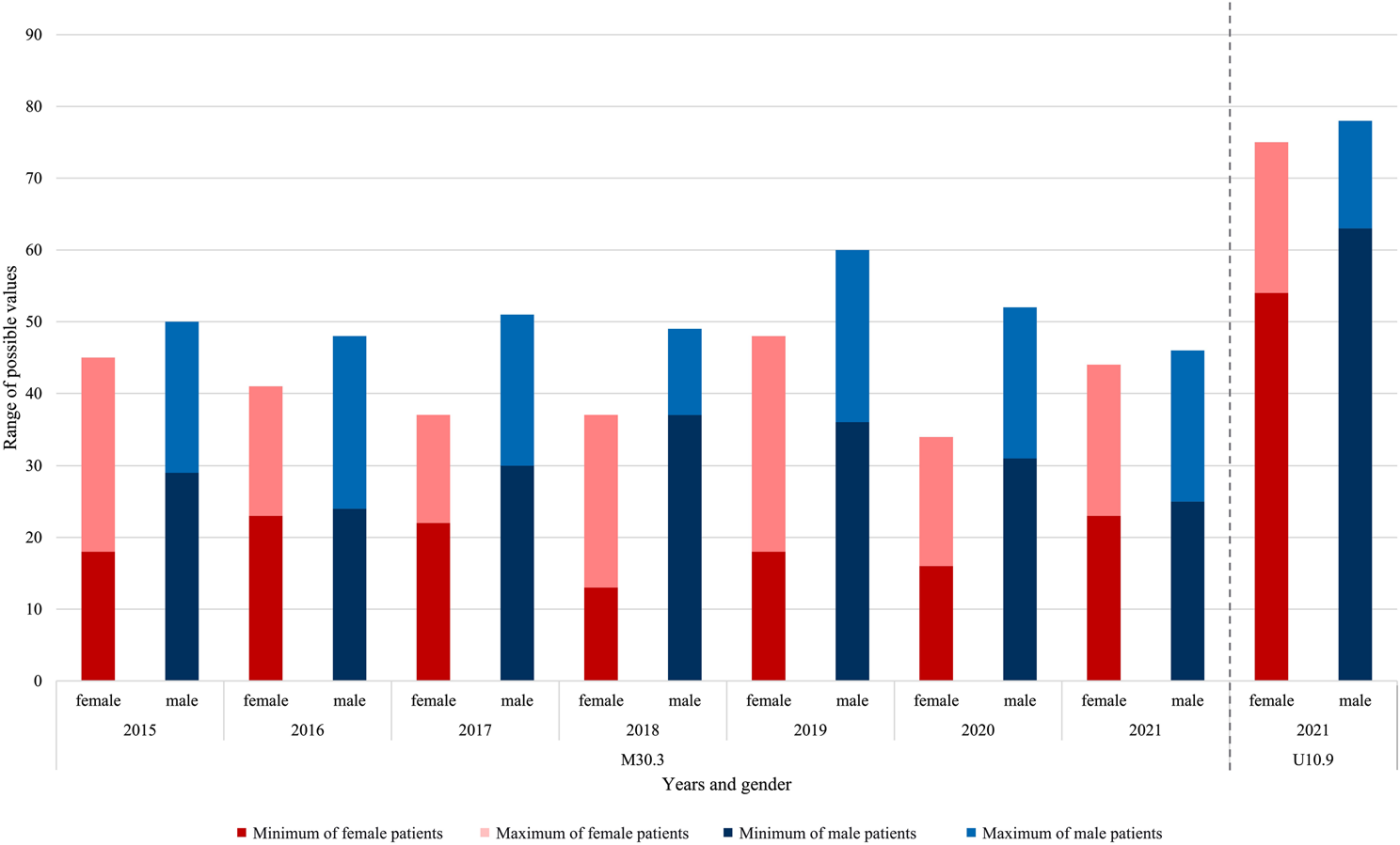
Range of possible values of patients aged 0–18 years with Kawasaki disease or MIS-C, by gender, based on aggregated anonymized inpatient data from participating German university hospitals, 2015–2021. ICD-10-GM codes: M30.3 = Kawasaki disease, U10.9 = MIS-C

Figure 4 shows descriptively that across all years and for both ICD codes, there is a higher prevalence of male children detected compared to female children (see Table 8 (additional file)).

#### Kawasaki disease and MIS-C depending on age groups (to answer research question 3d)

**Fig. 5:**
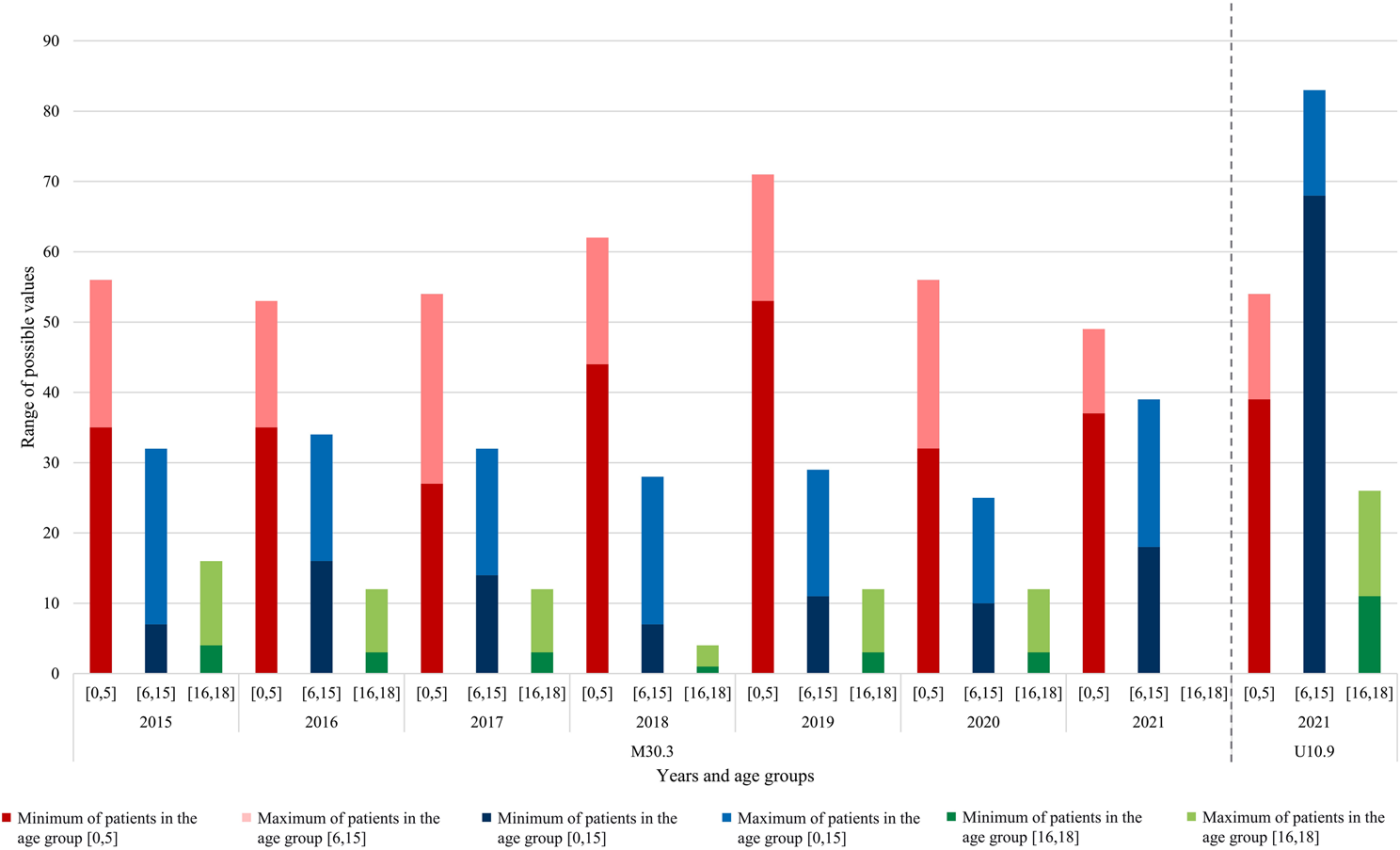
Range of possible values of patients aged 0–18 years with Kawasaki disease or MIS-C, by age group ([0,5], [6,15], [16,18]), based on aggregated anonymized inpatient data from participating German university hospitals, 2015–2021. ICD-10-GM codes: M30.3 = Kawasaki disease, U10.9 = MIS-C

When looking at the number of patients with Kawasaki disease and MIS-C across the three age groups, it was observed that the frequency of Kawasaki patients decreased in all age groups across all years (see Fig. 5). Conversely, the majority of MIS-C patients were observed within the age group of 6 to 15 years (see Fig. 5; see Table 9 (additional file)).

## Discussion

Despite valuable results, the research questions (see Table 1) could not be answered in their entirety. The reasons for this were, e.g., the incomplete exploitation of necessary data within the data integration centers (e.g. vital signs from neonatology). No inferential statistics were used because the descriptive analysis was already sufficient to identify and describe a trend in the data. In some cases, the anonymity of results data reduced the informative value and therefore no correlations could be calculated and the results were mainly shown descriptive as totals. Due to the anonymized and decentralized design, it cannot be excluded that patients treated at multiple participating hospitals during the observation period were counted more than once. This limitation is inherent to decentralized analyses without record linkage. If patient consent is available, privacy-preserving record linkage methods, such as the federated Trusted Third Party infrastructure [29], could be applied to address this issue in future studies.

Nevertheless, three decentralized studies on rare diseases from data of up to 17 German university hospitals could be conducted.

Based on the experience gained from conducting the three decentralized studies, hurdles in the secondary use of patient data in the context of rare diseases became apparent, which are discussed below.

### Use case (1): CF and pregnancy or delivery

The primary question (see Table 1) was to check whether the patients gave birth in a specialized CF center. Although this was not fully answered because we did not additionally ascertain whether the participating study sites also have a CF center, there are overlaps between the study results and the medical state of the art. When looking at CF patients and their complications during pregnancy and birth, the high proportion of gestational diabetes mellitus in CF (ICD code: O24.4) was striking. In the general European population, the prevalence of gestational diabetes is estimated to be about 7.8% [30]. However, our data are in line with a report on high prevalence rates of gestational diabetes mellitus in CF [31].

### Use case (2): PKU, comorbidities and pregnancy or delivery

The low incidence of neurological comorbidities in PKU can be explained by the success of newborn screening [32]. As the rare disease can now be diagnosed quickly, treatment can also be started quickly and brain damage be prevented. Nevertheless, adult PKU patients appear now more prone to depression; this is also indicated by recent studies [17, 32].

The available data show only a few complications during pregnancy or delivery in PKU patients. There is also no evidence of the maternal PKU syndrome [33]. This can also be explained by the fact that PKU is currently diagnosed quickly and is easily treatable as well as competent experience with pregnancy in PKU centers.

### Use case (3): Kawasaki disease and MIS-C

Based on the available data, results derived from MIS-C diagnosis since 2021, as the ICD code U10.9 was then introduced. An approximation using similar codes did not appear to be meaningful (see Table 5): For example, the number of cases for the codes of toxic shock syndrome, other shock, Systemic Inflammatory Response-Syndrome (SIRS) seemed too high. This means that the restriction to diagnosis codes from routine clinical documentation does not allow any statement to be made about MIS-C cases in 2020 – i.e. at the beginning of the COVID-19 pandemic.

**Table 5:** Range of possible values of patients with MIS-C and MIS-C-like diseases as well as Kawasaki disease in the observation period of 2025-2021

ICD code	Name of ICD code	Extreme value	2015	2016	2017	2018	2019	2020	2021
**M30.3**	Kawasaki disease	Minimum	68	62	56	58	76	49	63
		Maximum	74	71	74	73	85	70	72
A48.3, R57.8, R65*	Toxic shock syndrome, other shock, SIRS	Minimum	634	702	610	591	576	460	389
		Maximum	649	720	625	603	600	472	395
**D76.1–4**	Other specified diseases with participation of lymphoreticular and reticulohistiocytic tissue	Minimum	58	47	53	57	78	78	92
		Maximum	73	59	68	75	84	84	104
**D89.8/9**	Other specified disorders involving the immune mechanism	Minimum	5	4	7	7	24	20	21
		Maximum	20	16	28	28	39	32	36
**U10.9**	Multisystem inflammatory syndrome associated with COVID-19, unspecified	Minimum	-	-	-	-	-	-	128
		Maximum	-	-	-	-	-	-	140

An association between Kawasaki disease and MIS-C with positive COVID-19 findings could be confirmed, as indicated in a study conducted by the German Society for Pediatric Infectiology (German: “Deutsche Gesellschaft für Pädiatrische Infektiologie”; DGPI) examining MIS-C cases [34]. The study highlights the co-occurrence of Kawasaki disease and inflammatory syndromes, specifically MIS-C, a trend further supported by available data [34]. However, it is important to note that the decentralized nature of this study had limitations, as it solely considered diagnoses for COVID-19 findings and not laboratory SARS-CoV-2 test results. This omission may have resulted in information loss, given the absence of antibody tests. Interestingly, the DGPI study revealed a significant proportion of MIS-C patients with positive results in SARS-CoV-2 serology.

The available data from the decentralized study showed that males were more frequently affected by Kawasaki disease and MIS-C than females. Also, it is shown, that patients with Kawasaki disease are on average younger than MIS-C patients. This goes hand in hand with the results of the DGPI study and other studies on Kawasaki disease and MIS-C.

### Hurdles of secondary data use in the field of rare diseases

Various challenges arose during the conduction of the three decentralized studies based on secondary use of patient data. One challenge arose from specific regulations on the coding of diagnoses. For example, the use of ICD codes for the reimbursement of hospital services has an impact on the scope of ICD coding in the documentation. For example, mapping the cohort of Use Case 1 (CF and pregnancy or delivery) was difficult due to ICD regulatory, documentation guidelines and / or practice. It was not possible to document both diagnoses – CF and pregnancy or delivery – because both must be documented as primary diagnoses, but two primary diagnoses are not allowed. It was therefore necessary to first look for patients who had documented CF in their medical history in order to then search for pregnancies in these patients. However, this method led to an inflated count of pregnant CF patients at certain study sites. This overcounting occurred because CF diagnoses were sometimes recorded when a sweat test was conducted as a diagnostic measure, even if it did not confirm the suspicion of CF. Due to the insufficient documentation of diagnostic certainty, a selection process was necessary during cohort formation. It was crucial to ensure that patients had a minimum of two CF diagnoses in their clinical history to be unequivocally considered CF patients and accurately included in the cohort.

The comprehensive answering of research questions in Use Case 2 (PKU, comorbidities, and pregnancy or delivery) was hindered by the limitations of relying solely on routine data from inpatient stays at university hospitals. This approach may result in information loss, particularly regarding the (especially psychiatric) comorbidities of PKU patients, as these conditions are often addressed in outpatient settings and/or non-university hospitals rather than university hospitals. Although those additional diagnoses are often chronic diseases they have either not been entered into the diagnose list of the PKU patients when they present for their regular check-ups at the university departments or PKU was not listed by the psychiatry departments, when the patients were diagnosed there.

Similarly, in Use Case 3 (Kawasaki disease and MIS-C), there is a potential loss of information attributed to the availability of data from outpatient and inpatient care as well as to imprecise definition of cohorts. Notably, only diagnoses codes were considered, neglecting other crucial data elements such as laboratory findings. This oversight led to instances where ICD codes intended exclusively for pediatric diseases were applied in adult medicine, and certain diseases lacking specific ICD codes could not be considered, even when approximating other codes for similar diagnoses.

The challenges described in the three use cases are examples of the hurdles that can arise when using secondary health care data in the context of rare diseases at present. In general, the following hurdles were identified: (1) health care process bias, (2) inaccurate coding of ICD diagnoses, (3) difficult verification of results, (4) loss of information due to masking of small numbers of cases (in the present study due to applying a frequency threshold rule), (5) imprecise definition of the cohort.

1. Health care process bias describes a phenomenon where the process of patient health care – both with regard to the human component through the influence of doctors, nurses, relatives and patients [35] and with regard to legal regulation – impact the documentation of routine data in such a way that the observed data is distorted. This bias often leads to inaccuracy and incompleteness of the data [36], which in turn has an impact on the secondary data use. This bias should typically be recognized not as an issue of data quality, as indicated by Agniel et al. (2018) [37], but rather needs to be considered while analyzing the data. Using the example of the CF study (Use Case 1), it was possible to adapt the script to take into account the health care process bias by requiring separate documentation of CF and pregnancy or delivery as primary diagnoses, so that only confirmed diagnoses were included.
2. The inaccurate description of rare diseases by billing-relevant coding systems has already been discussed in detail in the literature [38, 39]. Classifications such as ICD-10, which are primarily used for billing or static evaluations and thus partially abstract from the actual medical concept, are often too imprecise for rare disease diagnoses. Additional terminologies offer more possibilities and can differentiate certain diagnosis more clearly, especially in the context of rare diseases. One opportunity are ORPHAcodes, which guarantee an unique identifier for each rare disease [40, 41]. At the time when this study was conducted, only individual university hospitals additionally documented rare diseases with ORPHAcodes, why decentralized analyses based on secondary data with ORPHAcodes were not possible. Only since spring 2023, coding using ORPHAcode and the German Alpha-ID-SE [42] has been mandatory for inpatients [43].
3. The definition of distributed analyses is that the secondary data of the participating sites are accessed and analyzed locally, so that only outcome data are reported back. This setup presents challenges in result verification, making it difficult to confirm, e.g., if the reported number of patients is accurate or if analytic errors arise from data integration inaccuracies or script programming issues. Different documentation habits or data modeling approaches among the different study sites processing the routine data can result in different characteristics of the resulting data. In the three conducted decentralized studies, despite a detailed description of the FHIR profiles in the form of implementation guides, there were instances of differing interpretations or inherent diversity in source data. Consequently, scripts had to undergo multiple rounds of testing at various locations, and interim results required verification by medical experts. The medical experts conducted a case-by-case review to assess the plausibility of data records for each use case, contributing to the refinement of selection criteria.
4. In order to avoid re-identification of patients with rare diseases, we applied a frequency threshold rule of 5; i.e. all values less than five and greater than zero were masked with the category ‘<5’. This resulted in uncertainty regarding the count of patients, with values ranging from 1 to 4. This also resulted in the ranges with possible values, as the maximum was calculated by multiplying the number of ‘<5’ by four. However, this procedure can lead to major distortions, especially in the case of rare diseases. The example of Use Case 2 (PKU, comorbidities and pregnancy or delivery) illustrates this phenomenon: When looking at comorbidities, no statement can be made as to whether F32.1 with a possible value range of [2–8] actually includes more patients than the diagnosis F32.8 with a possible value range of [1–4]. This means that only a trend can be assumed, but no statement can be made about the concrete frequencies found.
5. In some cases, the cohorts that were described medically are only inaccurately represented in the data. This is mainly due to the fact that the inclusion criteria were restricted to individual modules (e.g. diagnosis) and classifications (e.g. ICD-10-GM). This restriction was made for the present studies in order to take into account the partly different data sets of the participating data integration centers.

This list of challenges is not exhaustive. They need to be considered in the design, conduct, analysis and interpretation of studies based on secondary data in the field of rare diseases.

## Conclusions

As part of CORD-MI, three studies on rare diseases involving 14 to 17 German university hospitals were successfully conducted. This did not only effectively validate the distributed analysis process but also offered an insight into the healthcare landscape for rare diseases in Germany. Bringing attention to this specific medical domain raised awareness within the interdisciplinary project team: Medical experts gained an appreciation for the importance of data quality as well as peculiarity of secondary use of patient data, while data scientists developed an understanding of the medical aspects, which are important to consider when performing studies on rare diseases.

Although decentralized analyses have great potential, especially in the context of rare diseases. Data can be aggregated for as many patients as possible while preserving the privacy of sensitive patient data. Experience to date shows that there are still hurdles to overcome in distributed analyses of secondary health care data on rare diseases. In our previous work [12], we identified eight Lessons Learned from a medical informatics perspective for conducting decentralized studies based on secondary use of patient data. The present study builds on these methodological findings but extends them substantially by incorporating medical domain-specific Lessons Learned that only emerged through the in-depth analysis of the clinical use cases. By systematically addressing both the technical-methodological and the medical-domain-specific hurdles, the present work complements the previous findings by adding a focus on the medical validity, interpretability, and clinical applicability of decentralized secondary data analyses in rare diseases. Together, these perspectives provide a more complete view that supports the translation of methodological innovation into reliable and clinically meaningful research outcomes.

1. Minimizing the health care process bias requires domain knowledge, in particular by taking into account the documentation of routine data and the coding of rare diseases when using secondary data. Domain knowledge and intensive collaboration between medical experts and data scientists creates a basis for this.
2. The imprecise description of rare diseases can be counteracted by using additional terminology. This includes both the establishment in the clinical process (e.g., anamnesis and diagnosis) and in the documentation process as well as the exploitation of alternative coding for data modeling. It is as yet unclear how the mandatory coding using ORPHAcodes and Alpha-ID-SE can improve the quality of secondary use of patient data in Germany. Two aspects are particularly critical: On the one hand, the obligation to code for billing purposes and not as additional medical information can lead to distortion. Perhaps too many rare diseases will be documented due to the resulting monetary advantages. On the other hand, there is currently no obligation to use these terminologies in the outpatient sector, although most people with rare diseases do not receive inpatient care for their medical condition.
3. The challenging validation of analysis scripts and resulting data can be mitigated through personalized plausibility checks and iterative feedback loops with medical experts. Going forward, it would be beneficial to explore whether existing guidelines for secondary data analysis in public health / health care research and epidemiology [44, 45] can be adapted for clinical data processed by data integration centers in (university) hospitals. Another possibility for enhancing data interoperability involves the consistent application of standards. A unified and standardized database streamlines the development of analysis scripts. This could include the use of data and information models in the form of standardized research data repositories or detailed descriptions using data dictionaries or meta data repositories (e.g., Portal of Medical Data Models (MDM) [46] or ART-DECOR [47]).
4. To avoid the loss of information by masking small numbers – for example by applying frequency threshold rules – other privacy-preserving approaches can be adapted to the requirements of rare diseases. These methods include, e.g. Secure Multi-Party Computation (SMPC) or Personal Health Train (PHT) [48].
5. Approaches for Electronic Health Record (EHR)-driven phenotyping are helpful in order to map cohorts in secondary use of patient data as accurately as possible. EHR-driven phenotyping describes a process for determining medically relevant concepts of a target cohort based on raw data from the EHR [49]. How existing approaches can be applied in the context of rare diseases needs to be examined.

While challenges persist in the secondary use of data in the field of rare diseases, distributed analyses have yielded first prototype results. To enhance the quality and medical meaningfulness of these findings, it is crucial to adapt established methods and approaches to the requirements of this specific medical domain. By doing so, the complete potential of distributed analyses using secondary health care data can be unlocked. This aims on advancing research and enhancing medical care provided to patients with rare diseases.

## Electronic supplementary material

Below is the link to the electronic supplementary material.

Supplementary Material 1

## Data Availability

The dataset supporting the conclusion of this article is included within the article and its additional files. The additional information are available in the following repositories: • Study protocols: 10.5281/zenodo.10656436 [21]. • Formalized information: 10.5281/zenodo.10213532 [50]. • *R* scripts: - Cystic Fibrosis and pregnancy: https://github.com/medizininformatik-initiative/usecase-cord-support/tree/master/Studienprotokolle/CF. • Phenylketonuria, comorbidities and pregnancy: https://github.com/medizininformatik-initiative/usecase-cord-support/tree/master/Studienprotokolle/PKU. • Kawasaki disease and MIS-C: https://github.com/medizininformatik-initiative/usecase-cord-support/tree/master/Studienprotokolle/Kawasaki_PIMS. • Details of the *FDPG* projects: 10.5281/zenodo.11034586 [51].

## Abbreviations

BDSG: German Federal Data Protection Law (German: “Bundesdatenschutzgesetz”)
CORD-MI: Collaboration on Rare Diseases – Medical Informatics
DGPI: German Society for Pediatric Infectiology (German: “Deutsche Gesellschaft für Pädiatrische Infektiologie”)
EHR: Electronic Health Record
FHIR: Fast Healthcare Interoperability Resource
GDPR: General Data Protection Regulation
ICD-10-GM: German Modification of International Statistical Classification of Diseases and Related Health Problems in the 10th Version
MII: Medical Informatics Initiative
PHT: Personal Health Train
MIS-C: Multisystem Inflammatory Syndrome in Children
SIRS: Systemic Inflammatory Response-Syndrome
SMPC: Secure Multi-Party Computation
SNOMED CT: Systematized Nomenclature of Medicine Clinical Trials
SQL: Structured Query Language
