# Supplementary material for: Decentralized rare disease studies in Germany: first results and hurdles of secondary use of patient data
Source: BMC Med Inform Decis Mak. 2026 Aug 12;26:313. doi: 10.1186/s12911-026-03688-7 (PMC13471175; doi:10.1186/s12911-026-03688-7)
Supplement: Supplementary file 1 [file 12911_2026_3688_MOESM1_ESM.pdf]

Decentralized Rare Disease Studies in Germany: First Results and Hurdles of Secondary Use of Patient Data

Michele Zoch [1], Christian Gierschner [1], Jens Weidner [1], Martin Sedlmayr [1], Gabriele Müller [2], Daniela Choukair [3], Georg F. Hoffmann [3], Nicole Toepfner [4], Reinhard Berner [4], Fabian Prasser [5], Josef Schepers [5], Helge Hebestreit [6]

[1] Institute for Medical Informatics and Biometry at the Medical Faculty Carl Gustav Carus, TUD Dresden University of Technology, Germany

[2] Center for Evidence-based Healthcare, Medical Faculty and University Hospital Carl Gustav Carus, TUD Dresden University of Technology, Germany

[3] Center for Rare Diseases, University Hospital Heidelberg, Heidelberg, Germany

[4] University Centre for Rare Diseases and Department of Pediatrics, Medical Faculty and University Hospital Carl Gustav Carus, TUD Dresden University of Technology, Germany

[5] Berlin Institute of Health (BIH) at Charité Universitätsmedizin Berlin, Berlin, Germany

[6] Center for Rare Diseases – Reference Center Northern Bavaria, University Hospital, Julius-Maximilians University, Würzburg, Germany

Additional Files / Supplemental Tables

Table 1. Number of patients with CF in combination with diagnoses indicating pregnancy or childbirth in the observation period of 2015 – 2021 ____ 2

Table 2. Number of patients with CF and complications during pregnancy or delivery in the observation period of 2015 – 2021 ____ 3

Table 3. Number of patients with PKU and comorbidities in the observation period of 2015 – 2021 ____ 4

Table 4. Relative frequency of patients with PKU associated with internal, neurological or psychiatric disease depending on three age groups ([0,17], [18,30], [31,99]) in the observational period of 2015 – 2021 ____ 5

Table 5. Number of patients with classical PKU (E70.0) and complications during pregnancy or delivery in the observation period of 2015 – 2021 ____ 6

Table 6. Number of patients with other hyperphenylalaninaemias (E70.1) and complications during pregnancy or delivery in the observation period of 2015 – 2021 ____ 6

Table 7. Number of patients with Kawasaki disease or MIS-C in the observation period of 2015 – 2021 ____ 7

Table 8. Number of patients with Kawasaki disease or MIS-C depending on gender in the observation period of 2015 – 2021 ____ 8

Table 9. Number of patients with Kawasaki disease or MIS-C depending on three age groups [0,5], [6,15], [16,18] in the observation period of 2015 – 2021 ____ 9

USE CASE (1): CF, PREGNANCY AND DELIVERY

NUMBER OF PATIENTS WITH CF AND PREGNANCY OR DELIVERY

Table 1. Number of patients with CF in combination with diagnoses indicating pregnancy or childbirth in the observation period of 2015 – 2021

ICD code	Name of ICD code	Minimum	Maximum
**O09.1**	Duration of pregnancy: 5th to 13 completed weeks	4	16
**O09.2**	Duration of pregnancy: 14th to 19 completed weeks	8	14
**O09.3**	Duration of pregnancy: 20th to 25 completed weeks	4	16
**O09.4**	Duration of pregnancy: 26th to 33 completed weeks	19	31
**O09.5**	Duration of pregnancy: 34th to 36 completed weeks	11	20
**O09.6**	Duration of pregnancy: 37th to 41 completed weeks	10	19
**O09.7**	Duration of pregnancy: More than 41 completed weeks	2	8
**O09.9**	Duration of pregnancy: Unspecified	2	8
**O24.4**	Diabetes mellitus arising in pregnancy	22	55
**O30.0**	Twin pregnancy	9	12
**O63.0**	Prolonged first stage (of labor)	1	4
**O63.1**	Prolonged second stage (of labor)	1	4
**O64.0**	Obstructed labor due to incomplete rotation of fetal head	1	4
**O64.1**	Obstructed labor due to breech presentation	2	8
**O75.0**	Maternal distress during labor and delivery	1	4
**O80**	Spontaneous vertex delivery	4	16
**O80 Z37.0!**	Spontaneous vertex deliver within with the outcome of a single live birth	2	8
**O82**	Single delivery by caesarean section	3	12
**Z37.0**	Single live birth	20	26
**Z37.2**	Twins, both liveborn	1	4

NUMBER OF PATIENTS WITH CF AND COMPLICATIONS DURING PREGNANCY OR DELIVERY

Table 2. Number of patients with CF and complications during pregnancy or delivery in the observation period of 2015 – 2021

ICD code	Name of ICD code	Minimum	Maximum
**O24.4**	Diabetes mellitus arising in pregnancy	22	55
**O64.0**	Obstructed labor due to incomplete rotation of fetal head	1	4
**O64.1**	Obstructed labor due to breech presentation	2	8
**O75.0**	Maternal distress during labor and delivery	1	4
**O75.2**	Pyrexia during labor, not elsewhere classified	2	8

Use Case (2): PKU, Comorbidities and Pregnancy or Delivery

Number of patients with PKU and comorbidities

Table 3. Number of patients with PKU and comorbidities in the observation period of 2015 – 2021

**ICD code**	**Name of ICD code**	**Classical PKU** (E70.0)		**Other Hyperphenylalaninemia** (E70.1)	
		**Minimum**	**Maximum**	**Minimum**	**Maximum**
**F32.1**	Moderate depressive episode	2	8	2	8
**F32.8**	Other depressive episodes	1	4	1	4
**F32.9**	Depressive episode, unspecified	2	8	1	4
**F33.0**	Recurrent depressive disorder, current episode mild	1	4	0	0
**F33.1**	Recurrent depressive disorder, current episode moderate	2	8	2	8
**F33.2**	Recurrent depressive disorder, current episode severe without psychotic symptoms	1	4	1	4
**F33.4**	Recurrent depressive disorder, currently in remission	2	8	0	0
**F33.8**	Other recurrent depressive disorders	1	4	0	0
**G31.9**	Degenerative disease of nervous system, unspecified	0	0	1	4
**N18.1**	Chronic kidney disease, stage 1	1	4	0	0
**N18.2**	Chronic kidney disease, stage 2	1	4	1	4
**N18.3**	Chronic kidney disease, stage 3	1	4	1	4
**N18.4**	Chronic kidney disease, stage 4	0	0	1	4
**N18.5**	Chronic kidney disease, stage 5	0	0	1	4

NUMBER OF PATIENTS WITH PKU AND INTERNAL, NEUROLOGICAL OR PSYCHIATRIC DISEASE DEPENDING ON THREE DIFFERENT AGE GROUPS

Table 4. Relative frequency of patients with PKU associated with internal, neurological or psychiatric disease depending on three age groups ([0,17], [18,30], [31,99]) in the observational period of 2015 – 2021

**Age group**	**ICD code**	**Name of ICD code**	**Relative to all patients with Classical PKU** (E70.0)	**Relative to all patients with Other Hyperphenyl-alaninemia** (E70.1)
[0,17]	F32.1	Moderate depressive episode	0.54%	0.80%
	G31.9	Degenerative disease of nervous system, unspecified	-	0.20%
	N18.2	Chronic kidney disease, stage 2	0.25%	0.20%
	N18.3	Chronic kidney disease, stage 3	-	0.28%
	N18.5	Chronic kidney disease, stage 5	-	6.67%
[18,30]	F33.2	Recurrent depressive disorder, current episode severe without psychotic symptoms	1.82%	0.28%
[31,99]	F32.1	Moderate depressive episode	0.25%	0.20%
	F32.8	Other depressive episodes	0.25%	0.20%
	F32.9	Depressive episode, unspecified	1.77%	0.20%
	F33.0	Recurrent depressive disorder, current episode mild	0.34%	-
	F33.1	Recurrent depressive disorder, current episode moderate	0.30%	0.35%
	F33.4	Recurrent depressive disorder, currently in remission	16.67%	-
	F33.8	Other recurrent depressive disorders	3.03%	-
	N18.1	Chronic kidney disease, stage 1	0.34%	-
	N18.3	Chronic kidney disease, stage 3	0.25%	-
	N18.4	Chronic kidney disease, stage 4	-	0.80%
	N18.5	Chronic kidney disease, stage 5	-	0.24%

NUMBER OF PATIENTS WITH PKU AND COMPLICATIONS DURING PREGNANCY OR DELIVERY

Table 5. Number of patients with classical PKU (E70.0) and complications during pregnancy or delivery in the observation period of 2015 – 2021

ICD code	Name of ICD code	Minimum	Maximum
**O24.4**	Diabetes mellitus arising in pregnancy	2	8
**O64.1 Z37.0!**	Obstructed labor due to breech presentation with single live birth as outcome of delivery	1	4
**O64.8**	Obstructed labor due to other malposition and malpresentation	1	4
**O75.7**	Vaginal delivery following previous caesarean section	1	4

Table 6. Number of patients with other hyperphenylalaninaemias (E70.1) and complications during pregnancy or delivery in the observation period of 2015 – 2021

ICD code	Name of ICD code	Minimum	Maximum
**O24.4**	Diabetes mellitus arising in pregnancy	1	4

USE CASE (3): KAWASAKI DISEASE AND MIS-C

NUMBER OF PATIENTS WITH KAWASAKI DISEASE OR MIS-C IN 2015 TO 2021

Table 7. Number of patients with Kawasaki disease or MIS-C in the observation period of 2015 – 2021

ICD code	Name of ICD code	Extreme value	2015	2016	2017	2018	2019	2020	2021
**M30.3**	Kawasaki disease	Minimum	68	62	56	58	76	49	63
		Maximum	74	71	74	73	85	70	72
**U10.9**	Multisystem inflammatory syndrome associated with COVID-19, unspecified	Minimum	-	-	-	-	-	-	140
		Maximum	-	-	-	-	-	-	128

Number of patients with Kawasaki disease or MIS-C depending on gender

Table 8. Number of patients with Kawasaki disease or MIS-C depending on gender in the observation period of 2015 – 2021

ICD code	Name of ICD code	Gender	Extreme value	2015	2016	2017	2018	2019	2020	2021
**M30.3**	Kawasaki disease	female	Minimum	18	23	22	13	18	16	23
			Maximum	45	41	37	37	48	34	44
		male	Minimum	29	24	30	37	36	31	25
			Maximum	50	48	51	49	60	52	46
**U10.9**	Multisystem inflammatory syndrome associated with COVID-19, unspecified	female	Minimum	-	-	-	-	-	-	54
			Maximum	-	-	-	-	-	-	75
		male	Minimum	-	-	-	-	-	-	63
			Maximum	-	-	-	-	-	-	78

NUMBER OF PATIENTS WITH KAWASAKI DISEASE OR MIS-C DEPENDING ON AGE

Table 9. Number of patients with Kawasaki disease or MIS-C depending on three age groups [0,5], [6,15], [16,18] in the observation period of 2015 – 2021

ICD code	Name of ICD code	Age group	Extreme value	2015	2016	2017	2018	2019	2020	2021
**M30.3**	Kawasaki disease	[0-5]	Minimum	35	35	27	44	53	32	37
			Maximum	56	53	54	62	71	56	49
		[6-15]	Minimum	7	16	14	7	11	10	18
			Maximum	32	34	32	28	29	25	39
		[16,18]	Minimum	4	3	3	1	3	3	0
			Maximum	16	12	12	4	12	12	0
**U10.9**	Multisystem inflammatory syndrome associated with COVID-19, unspecified	[0-5]	Minimum	-	-	-	-	-	-	39
			Maximum	-	-	-	-	-	-	54
		[6-15]	Minimum	-	-	-	-	-	-	68
			Maximum	-	-	-	-	-	-	83
		[16,18]	Minimum	-	-	-	-	-	-	11
			Maximum	-	-	-	-	-	-	26
